# Anxiety, depression, somatization and psychological distress before and 2–6 years after a late termination of pregnancy due to fetal anomalies

**DOI:** 10.1186/s12905-024-03082-3

**Published:** 2024-04-24

**Authors:** Lisa Irmscher, Romy Marx, Maike Linke, Anja Zimmermann, Stephanie Drössler, Hendrik Berth

**Affiliations:** 1 Medizinische Fakultät Carl Gustav Carus, Psychosoziale Medizin und Entwicklungsneurowissenschaften, Forschungsgruppe Angewandte Medizinische Psychologie und Medizinische Soziologie, Technische Universität Dresden, 01307 Dresden, Germany; 2 Charité Universitätsmedizin Berlin, Referat für Studienangelegenheiten, 10115 Berlin, Germany; 3 Freistaat Sachsen, Landesamt für Schule und Bildung, Beratungsstelle zur Begabtenförderung, 01445 Berlin, Germany

**Keywords:** Late termination of pregnancy, Late term abortion, Pregnancy termination, Anxiety, Depression, Somatization, Psychological distress

## Abstract

**Background:**

For many women, a late termination of pregnancy (TOP) can be an enormous psychological burden. Few studies have investigated the long-term psychological impact of late TOP.

**Methods:**

*N* = 90 women answered a questionnaire containing questions about anxiety, depression and somatization (Brief-Symptom Inventory, BSI-18) shortly before (T1) and 2–6 years after (T4) their late termination of pregnancy.

**Results:**

Prior to the late TOP, 57.8% of participants showed above-average levels of overall psychological distress (66.7% anxiety, 51.1% depression, 37.8% somatization). This number decreased significantly over time for all scales of the BSI-18. 2–6 years later, only 10.0% of women still reported above-average levels (17.8% anxiety, 11.1% depression, 10.0% somatization).

**Conclusions:**

Our results support those of previous research showing that late TOP has a substantial psychological impact on those experiencing it in the short-term. In the long-term, most women return to normal levels of psychological distress, although some still show elevated levels. Limitations of the study include monocentric data collection, drop-out between T1 and T4, and the relatively wide range of two to six years after TOP. Further research should be conducted in order to identify factors that impact the psychological processing of the experience.

## Introduction

As much as terminations of pregnancy (TOP) are discussed, often those discussions are not focused on the lived experiences of the people in need of one, specifically their psychological well-being. A particular case of TOP with a different psychological impact are late TOP [1]. The term “late termination of pregnancy” or “late-term abortion” is usually used in reference to abortions after the 12th week or first trimester of pregnancy, although there is no clear medical definition [2]. This definition is based on the German law, whereby abortions during the first twelve weeks of pregnancy are exempt from punishment (albeit being illegal) if certain guidelines are followed [3]. After the 12th week, the pregnancy may only be terminated if there is a serious danger to the life, physical or mental health of the pregnant person and there is no other reasonable way to prevent it [3]. This encapsulates TOP as a result of lethal fetal anomalies and pregnancies that place an insufferable psychological burden on the pregnant person, i.e. due to non-lethal anomalies in the child or the young age of the mother. In 2022 there were 103,927 terminations of pregnancy in Germany, 3,113 (3.0%) of which were TOP after the 12th week of pregnancy [4].

Studies investigating regular TOP could not find a link between the TOP and short-term or long-term psychological distress for most participants [5, 6]. This seems to be different for late TOP as the experience is oftentimes traumatic and evokes feelings of grief [7, 8]. Coleman et al. found that participants reported more posttraumatic stress symptoms after a late TOP compared to a TOP in the first twelve weeks [1]. Similarly, Davies et al. [9] compared first trimester TOP to second trimester TOP due to fetal anomaly. The participants with late TOP showed significantly more post-traumatic stress symptoms six weeks after TOP. Moreover, they found high levels of distress in both groups six weeks, six months and twelve months after TOP. These results resemble those of other studies that show high levels of psychological distress shortly after late TOP [10–14]. However, it is not clear how long it takes for the psychological distress and symptoms of posttraumatic stress to return to a normal range. Iles and Grath [10] report near normal levels of psychiatric morbidity six and twelve months after late TOP, similar to Güçlü et al. [14], who examined grief at the same intervals. Götzmann et al. found normal levels of psychological distress just four weeks after late TOP [15]. In other studies, 46% of participants showed pathological levels of posttraumatic stress four months after TOP, 21% 16 months after TOP [11] and 17% two to seven years after TOP [13]. For depression, the figures were 28% after four months and 13% after 16 months [11]. Furthermore, Kersting et al. found that 14 months after late TOP participants could be diagnosed with psychiatric disorders more often than participants who had a preterm birth or a regular birth [12]. In a study from 2018, 18.9% of women reported above-average levels of posttraumatic stress, 17.5% had a so-called complicated grief reaction and 10.8% showed elevated levels of depression one to seven years after their late TOP [16]. Examining the same sample that was used in the present study, Zimmermann et al. found that 21% of participants still reported significant signs of psychological distress and 28% reported significant levels of posttraumatic stress on average 22 months after late TOP [17].

The studies to date all show a decline of symptoms of distress over time but are heterogenous regarding the duration of the symptoms. Only very few of them have examined the psychological impact over a time span longer than two years. Therefore, the current study aims to contribute to the question of whether levels of psychological distress stay elevated two to six years after late TOP. It is hypothesized that psychological distress decreases over time and only few women still show increased psychological distress two to six years after a late TOP.

## Methods

### Sample

In this longitudinal study, data was collected at four points in time: before a late TOP (T1), six months after TOP (T2), two years after TOP (T3) and approximately four years (*M* = 4.16, *SD* = 1.22, range = 2–6) after TOP (T4). At T4, 11.1% of the participants had terminated their pregnancy two years ago, 21.1% three years ago, 26.7% four years ago, 21.1% five years ago and 20.0% six years ago. The surveys T2 and T3 are not the subject of the analyses presented here.

The sample for T1 comprised of *n* = 160 women, *n* = 90 (56.3%) of which were still part of the sample at T4, resulting in a total sample size of *n* = 90. The sample was recruited from patients who were referred to the University Hospital Carl Gustav Carus in Dresden, Germany for a psychological consultation due to a pathological fetal finding, as required by German law [3]. Women under 18 years, women that did not give written consent to participate in the study, and women who were not fluent in German were excluded from the sample. Data collection was conducted from June 2013 until July 2017 and from July 2019 until October 2019 for T1 and T4 respectively. The T1 survey was conducted as a paper-and-pencil questionnaire immediately before the psychological consultation. The follow-up survey T4 took place as an online survey using the platform Lime Survey. All participants in the first survey T1 were invited by letter to participate in the follow-up survey T4 up to three times. No statements can be made about the reasons for non-participation in T4. Participants did not receive a compensation fee.

The participant’s mean age at the start of data collection was 32 years (*M* = 32.31, *SD* = 5.52, range = 20–43). 43.3% of participants were married and 55.7% already had one or more children at T1. The mean pregnancy termination date was during the 18th week of pregnancy (*M* = 18.66, *SD* = 3.69, range = 12–29). The most common reasons for late TOP were chromosomal defects (47.2%) as well as complex malformation syndrome and anomalies of the central nervous system (30.3%). Of those fetal anomalies, 35.6% were highly likely to be lethal. 93.3% of the pregnancies were wanted.

### Questionnaire

Patients answered a comprehensive set of questions about sociodemographics (e.g. age, length of relationship, number of children) and pregnancy (e.g. gestational age, lethality of the diagnosis) as well as the Brief Symptom Inventory (BSI-18 or Mini-SCL), among others that will not be analyzed in this study.

The BSI-18 questionnaire is an abbreviated form of the symptom checklist SCL-90-R [18], it includes 18 items on psychological distress [19, 20]. Participants are asked to report symptoms of anxiety, depression and somatization that they may have experienced in the past seven days. Six items each are assigned to the scales Anxiety (e.g., “fright or panic attacks”), Depression (e.g., “feelings of loneliness”), and Somatization (e.g., “heart and chest pain”). An overall score for psychological distress (“Global Severity Index”, GSI) is calculated as the sum of all items. The answers are given on a five-point Likert scale from “not at all” (0) to “very strongly” (4). Higher values represent greater psychological distress. The range of values is 0 to 24 for the subscales and 0 to 72 for the total GSI.

Reliability (McDonald’s Omega) in the present study was: depression *ω* = 0.766 (T1), *ω* = 0.907 (T4), anxiety *ω* = 0.780 (T1), *ω* = 0.773 (T4), somatization *ω* = 0.702 (T1), *ω* = 0.790 (T4) and overall psychological distress (GSI) *ω* = 0.872 (T1), *ω* = 0.908 (T4).

### Statistical analysis

Statistical analyses were performed using IBM SPSS Statistics (version 29.0). Paired t-tests as well as independent sample t-tests and Pearson correlations were calculated. A significance level of *α* = 0.05 was chosen. Effect sizes were calculated as Cohen’s *d*. Population-representative, age- and gender-specific t-score standard values from the manual [19] were used to identify above-average scores on the BSI-18 scales (t-scores of 60 or higher are considered above average on all subscales).

The sample size was tested using G*Power 3.1.3 [21]. For the comparison of two groups by means of paired t-tests, a sample of at least *n* = 54 participants is necessary assuming a significance level of *α* = 0.05, an effect size of Cohen’s *d* = 0.5, and a power of 95% (1 - *β* = 0.95).

The present study was conducted in accordance with the Declaration of Helsinki. The study was approved by the Ethics Committee of the Technische Universität Dresden, Germany (EK253072013), and only individuals who provided written informed consent were included as study participants.

## Results

To check whether there was a systematic drop out of participants between T1 and T4, t-tests were conducted. There was no significant difference in age (*t*(158) = -0.796, *p* > .05), gestational age (*t*(158) = -0.716, *p* > .05) and scores on psychological distress scales (anxiety *t*(158) = -0.398, *p* > .05; depression *t*(127,670) = 0.943, *p* > .05; somatization *t*(129,383) = -0.641, *p* > .05; overall psychological distress *t*(158) = 0.469, *p* > .05) comparing participants who answered the questionnaire at both time points (*n* = 90) to participants who did not participate at T4 (*n* = 70).

As shown in Table 1, there was a strong, significant decrease in psychological distress over time on all scales of the BSI-18. Effect sizes were in the large range. Compared to age- and gender-specific standard values, many participants reported above-average scores of anxiety (66.7%, *n* = 60), depression (51.1%, *n* = 46), somatization (37.8%, *n* = 34) and overall psychological distress (57.8%, *n* = 52) at T1. At T4, these numbers decreased to 17.8% (anxiety, *n* = 16), 11.1% (depression, *n* = 10), 10.0% (somatization, *n* = 9) and 10.0% (psychological distress, *n* = 10).

**Table 1:** Anxiety, depression, somatization and overall psychological distress (GSI) before and 2–6 years after TOP (*n* = 90) (M, SD, paired t-tests, effect size Cohens d)

	Before TOP		2–6 years after TOP				
BSI-18	M	SD	M	SD	Test statistics	Significance	Effect size
Anxiety	6.33	4.49	1.84	2.39	T = 9.624 df = 89	*p* < .001	d = 1.014
Depression	6.10	4.24	1.91	3.10	T = 9.147 df = 89	*p* < .001	d = 0.964
Somatization	4.00	3.40	1.39	2.60	T = 7.080 df = 89	*p* < .001	d = 0.746
Overall Psychological Distress (GSI)	16.43	10.28	5.14	7.03	T = 10.439 df = 89	*p* < .001	d = 1.100

It was also examined whether there was a connection with sociodemographic variables. Pearson correlations of overall psychological distress with age (*r* = .20, *p* > .05), gestational age (*r* = − .10, *p* > .05) and length of relationship (*r* = − .14, *p* > .05) were not significant. The t-tests for lethality of the diagnosis (*t*(87)= -1.093, *p* > .05) and already having one or more children (*t*(48,922) = -1,183, *p* > .05) were not significant either.

## Discussion

Only few studies have examined the psychological impact of a late TOP over a longer time span. This study aims to contribute to the research field of how long-lasting the resulting psychological distress of a late TOP actually is. To this end, we analyzed data collected two to six years after a late TOP due to fetal anomalies.

We found a significant decrease in psychological distress on all scales (anxiety, depression, somatization, overall psychological distress) over time. More than half of women reported overall psychological distress in the above average range before their late TOP. Two to six years later, only 10% of women did. For anxiety, depression and somatization the numbers decreased at a similar rate. This confirms our hypothesis that psychological distress decreases over time and only few women still show increased psychological distress in the long-term. There were no significant results regarding a potential connection of psychological distress and sociodemographic factors (age, gestational age, length of relationship, lethality of the diagnosis, prior children).

There could be many reasons for the decline in psychological stress over time. The first survey was carried out in the acute, stressful phase of the decision before the TOP. Two to six years after the decision, most women have found a way to cope with the stressful event. This could be supported by the partner, the family or by professional help such as psychotherapy. The importance of the event in the women’s lives might simply have decreased over time alone. The birth of a healthy child could also help to cope with the event.

The results support the well-substantiated assumption that a late TOP is a highly stressful situation that can take a toll on mental health in the short-term [1, 7–14]. In the long-term, psychological distress decreases for most participants and only few women still show elevated levels. Korenromp et al. examined participants in a similar time span (2–7 years) and found that 17.3% still showed above average symptoms of posttraumatic stress [13]. Hanschmidt et al. reported that 1–7 years after late TOP 18.9% of women showed above average levels of posttraumatic stress, 17.5% had a complicated grief reaction and 10.8% showed elevated levels of depression [16]. In comparison, our results fit those of Korenromp et al. and Hanschmidt et al. [13, 16]. Other studies only looked at shorter time spans but also found a decrease in psychological distress, albeit smaller in size [11, 17]. However, some studies found that psychological distress returned to normal levels as early as after a few weeks or months [10, 14, 15]. This finding is not supported by our study.

In this study, we could not find a significant association between any sociodemographic variables and psychological distress. Prior studies in contrast found that higher gestational age and non-lethal fetal anomalies were associated with higher psychological distress [9, 11, 13]. Those differing results might be due to some of the limitations of our study as seen below. Further research is necessary to determine the influence of these factors. Maternal age and prior children did not have a significant effect on psychological distress [11]. This is supported by the results of our study.

### Limitations

The following limitations should be considered when interpreting the results of this study: The sample was recruited monocentrically at one German university hospital. Only 54.2% of the original sample could be included in the repeat survey T4. Although we did analyze the data for systematic drop out, there could be other factors not included in this study that were influencing the decision not to participate. Furthermore, no data was collected on the participant’s mental health prior to the fetal diagnosis, which may influence the processing of the TOP. During the long observation period of two to six years, other important factors may have influenced the psychological well-being of the participants. These include life events such as separation from a partner, the birth of children, but also starting psychotherapy. This cannot be adequately controlled for in a study of this kind. Furthermore, the range of two to six years after TOP is quite wide and thus does not allow for very specific results regarding the duration of distress symptoms. The BSI-18 is an internationally used instrument with excellent psychometric criteria. However, it is a relatively short screening instrument and not a detailed diagnostic interview.

## Conclusion

The most severe impact of a late TOP seems to be in the short-term, after which it takes months to years to reduce feelings of distress for some women. Other factors that have not been examined here may contribute to the fact whether women experience long-lasting psychological distress after a late TOP, e.g. prior psychiatric disorders, social support or previous TOPs and miscarriages. Those factors should be assessed in further research.

The psychological distress of women after late TOP will continue to be an important area of research, as new methods of prenatal diagnosis may also influence the decisions of women and couples. This is especially important in order to provide adequate support for those experiencing long-term mental health effects.

## Data Availability

The datasets generated and/or analyzed during the current study are available from the corresponding author on reasonable request.
